# Application of effective microorganisms for Littoral zone restoration in eutrophic reservoirs

**DOI:** 10.1038/s41598-025-01795-5

**Published:** 2025-05-14

**Authors:** Paweł Tomczyk, Barbara Wróbel, Czesława Rosik-Dulewska, Alban Kuriqi, Mirosław Wiatkowski, Witold Skorulski, Tomasz Kabat, Mirosław Prycik, Jarosław Drobnik, Łukasz Gruss, Andrzej Kłos

**Affiliations:** 1 Institute of Environmental Engineering, Wrocław University of Environmental and Life Sciences, 50-363 Wrocław, Poland; 2 Institute of Technology and Life Sciences, National Research Institute, 05-090 Falenty, Poland; 3 Institute of Environmental Engineering of the Polish Academy of Sciences, Zabrze, 41-819 Zabrze, Poland; 4 Civil Engineering Department, University for Business and Technology (UBT), 10000 Pristina, Kosovo; 5 Art Strefa Witold Skorulski, 51-604 Wrocław, Poland; 6 DATII (Dolnośląski Akcelerator Technologii i Innowacji) , Długołęka, Poland; 7 Department of Family Medicine, Wroclaw Medical University, Wrocław, Poland; 8 Institute of Environmental Engineering and Biotechnology, University of Opole, 45-032 Opole, Poland

**Keywords:** Bioremediation, Eutrophication, Sustainable development goals, Turawa reservoir, Water protection, Environmental sciences, Pollution remediation

## Abstract

Inland waters play an important ecological, social, and economic role. However, they are exposed to various types of pollution, mainly from agriculture, industry, and urbanization. Therefore, it is important to take measures to restore their biological balance by supporting the natural processes of bioremediation. One of the methods for such measures is the use of effective microorganisms (EM). The objectives of this study are therefore: (i) to verify the temporal and spatial variability of the results of microbiological parameters (total heterotrophic bacteria - HBN, microscopic fungi - MF, coliform count - CI, dehydrogenase activity - DHA) in the sandy littoral substrate (beach) collected within the eutrophic Turawa reservoir (Southern Poland, Central Europe); ii) comparison of the results of microbiological parameters at control points and during the application of EM (spraying the shore surface with a liquid bio-preparation); iii) verification of the effectiveness of EM on the microbiological parameters of the substrate collected in the coastal zone of the reservoir and comparison with the results from other studies. The statistical analyses performed (PCA, HCA, correlation matrix) showed a high relationship and correlation (R from 0.88 to 0.92) between the study points and discrepancies between the parameters tested. Statistical significance was demonstrated for CI when the group of control points was compared with EM application - there was an average decrease in CI of 48% after EM application (decrease from 6.31 · 10^−3^ g to 3.28 · 10^−3^ g). The results obtained were consistent with the literature for HBN and MF (an increase of 14.74 and 10.81 medians in the group with EM application; 0.07045 · 10^6^ CFU/g and 0.205 · 10^3^ CFU/g, respectively) and differed for DHA (decrease, marginal difference, i.e. 2.41%; 41.5 mg TPF/kg·h). The results described represent one of the case studies related to the bioremediation of water reservoirs and the improvement of sanitary safety in the vicinity of water reservoirs. The research fits into strategies for rational land management governed by numerous national and international legal acts, strategies, and policies.

## Introduction

Inland waters play an important ecological, social, and economic role^1^. However, the areas directly adjacent to them (i.e., soils, substrates) are exposed to various types of pollution, mainly from agriculture (e.g., fertilizers, pesticides), industry (e.g., oils, leachate), and urbanization (e.g., improper drainage, improper waste storage)^2,3^. It is therefore becoming increasingly important to take measures to protect these areas. In the European Union (EU), for example, the EU Soil Strategy for 2030 is in force, which refers, among other things, to the sustainable management of soils, the maintenance of their proper condition or adaptation to climate change^4,5^.

Protecting soils and substrates also involves cleaning them of pollutants^6,7^. These activities include a range of remediation methods based on two main mechanisms: (1) transfer of contaminants from the solid phase to the soil solution and (2) immobilization of contaminants in the solid phase (or a combination of both mechanisms)^8^. These methods can be applied in situ (cleaning at the site of contamination) or ex-situ (cleaning outside the contaminated site)^9^. The methods can be divided into the following types: Solidification, vitrification, extraction of contaminated soil (substrate), chemical immobilization, and bioremediation^10^.

The last type of method, i.e., bioremediation, also involves the use of effective microorganisms (EM), which use a selected mixture of bacteria, actinomycetes, yeasts, and fungi^11^. They act synergistically and restore the appropriate ecological balance of the ecosystem through the dominance of effective species in the soil^12^. Higa first researched this technology in Japan in the 1980 s. In 1991, the first results of this work were published, relating to the application of soil and foliar EM in horticulture^13^. To date, the scope of application of this technology has been expanded and tested in water purification in reservoirs and rivers, wastewater treatment, composting, medicine, animal husbandry, forestry, and agriculture, among others^14–16^.

Remarkable results following the application of EM in lakes and rivers include: (i) the reduction of about 80% of microcystins in the eutrophic lakes Tsukui and Sagami in Japan^17^; (ii) the reduction of nitrogen and phosphorus compounds — important factors for eutrophication — in water bodies in Hungary, India^18^ and Poland^19^; and (iii) the decrease of biochemical oxygen demand (BOD), chemical oxygen demand (COD) and ammonium nitrogen (NH₄-N) in river systems^20^. However, some studies have reported limited or no significant improvements in certain parameters after EM application: (i) EM treatments have not consistently reduced heavy metal concentrations^21^, (ii) increased dissolved oxygen levels^22^, or (iii) reduced turbidity in reservoirs^23^. Therefore, further comprehensive studies are needed to assess the extent and effectiveness of EM technology in surface water remediation^24^. One such example is the Turawa Reservoir, which is the subject of this article. Previous articles on the application of EM to the water of this reservoir^16,25^ demonstrated a 7.78 improvement in the trophic status of the Turawa Reservoir as expressed by the Carlson Index, which was reflected in the reduction of unfavorable microorganisms (HBN22, HBN36, CBN, FCBN, FEN) after the application of EM. A reduction in nitrogen and phosphorus compounds was also observed compared to the control samples after the application of biopreparations. The effect of EM itself was rather short-term (17.6–34.1 days) and should be improved.

The research on EM in soil was mainly concerned with testing the possibilities of its practical application in horticulture and agriculture. In particular, the aim was to test the effects of the application of EM on the yields and growth conditions of crops (e.g., wheat, beans, cucumbers, tomatoes, potatoes, onions, peppers)^26–30^, its effectiveness in combination with other fertilizers in the context of improving the functional properties of soils (e.g., compost, biochar) (e.g., compost, biochar, zeolites, manure, nitrogen fertilizers)^31–35^ or in relation to the nutrient content in plants after the application of EM (e.g. tomatoes, kiwi, beans)^37–39^. Some soil studies also referred to the use of EM as a method to clean pollutants - among others, the following were investigated: their potential to remove diflufenican and flurochloridone^40^, imidacloprid^41^, biodegradation of petroleum hydrocarbons^42^. Studies have also been conducted on the general condition of the soil after EM application, including physical and chemical properties, microbiological activity, the occurrence of pathogens and soil diseases, nutrient content, and air-water properties^43–47^. The literature review shows that most studies on EM relate to agricultural soils (i.e., with appropriate properties, including fertility). At the same time, fewer analyses are looking at soils and substrates with other properties, such as those near water reservoirs, as in the case of this work (these generally focused on examining the effects of EM on changes in water or soil quality, not on the intervening substrate zone in the littoral zone). Few studies have also looked at the microbiological properties of soils and substrates, which are the focus of this work.

The main objectives of this study are: (i) to examine the temporal-spatial variability of the results of microbiological parameters (i.e., the total number of heterotrophic bacteria - HBN, number of microscopic fungi - MF, number of coliforms - CI, dehydrogenase activity - DHA) in the substrate collected in the eutrophic Turawa Reservoir (Southern Poland, Central Europe) on selected dates in 2019 and 2020; ii) to compare the results of the microbiological parameters of the substrate at control points and when applying EM (spraying the substrate surface with a liquid bio-preparation); iii) to verify the effectiveness of EM on the microbiological parameters of the substrate collected near the Turawa reservoir (taking into account the data of EM application) and to compare the results obtained by other researchers. As has already been shown, EM can be a potential method of bioremediation in eutrophic reservoirs that can have a positive impact on water quality by using a mixture of microorganisms that do not pollute this ecosystem (unlike, for example, chemical methods that can radically disrupt its balance)14, so it was decided to verify this effectiveness also in relation to the substrate in the immediate vicinity of the reservoir (shoreline). This research is part of a project to introduce an innovative method of water treatment in eutrophic reservoirs using ion exchange resins. EM has been tested as one of the complementary methods to restore the reservoir and improve its trophic status (including testing the improvement of substrate properties in the littoral zone of the reservoir). The work fits in with international strategies and policies for soil conservation and the Sustainable Development Goals. It may be relevant as a case study for the use of EM in soils in relation to the possible changes in microbiological properties characterized by different parameters.

## Materials and methods

### Field research

**Fig. 1:**
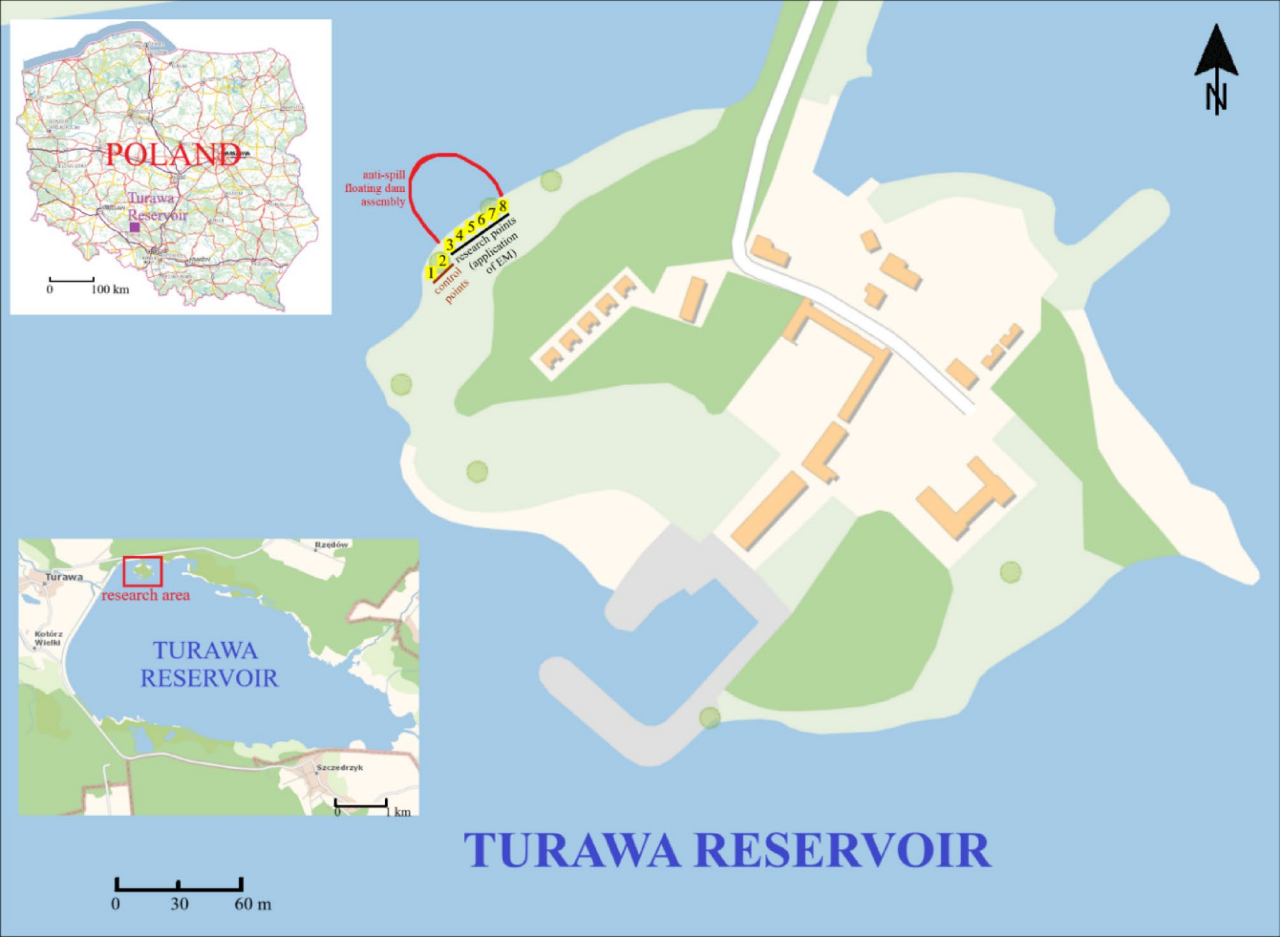
Location of the research area and sampling of the substrate within the Turawa reservoir, southern Poland (created in QGIS Desktop 3.34.9; map background: https://mapy.geoportal.gov.pl/imap/Imgp_2.html?gpmap=gp0).

The artificial Turawa reservoir, which is located on the Mała Panew River in southern Poland and has been in operation since 1939, was selected as a research site. It is used for retention, flood protection, energy supply, tourism (bathing areas, fishing spots), and nature (bird and fish sanctuary)^40^. Substrate samples (loose sand) weighing approx. 600 g each were taken from the surface layer (0–20 cm) in the north-western part of the reservoir in 2019 and 2020 using Egner’s soil rod. Eight study points were selected, including two control points (points 1–2) and 6 points covered by the application of effective microorganisms (EM; points 3–8) in the coastal zone at the floating FINBOOM dam (750 m^2^, which was created to separate the studied zone without water exchange from the rest of the reservoir, in the area recommended by the manager of the Turawa reservoir (area excluded from navigation)14,23. Sampling was repeated three times at each point. Their locations are shown in Fig. 1.

The substrate sampling dates were as follows: 2. July 2019 - control date; September 16, 2019 - one month after the application of the EM preparation; November 20, 2019 - three months after the application of the effective microorganisms; July 28, 2020 - one month after the application of the effective microorganisms, September 16, 2020 - one month after the application of the EM (was not carried out due to the high water level in the reservoir and the flooding of the coastal zone) and November 23, 2020 - one month after the application of the effective microorganisms.

EM was applied to the substrate in the form of a liquid solution using a handheld backpack sprayer on the soil surface on the following dates: August 2019, June 2020, August 2020, and October 2020. The initial solution of the ProBiosanit preparation (250 l of microorganism cultures + 100 l of plasma water) with water at a ratio of 1:10 was used at a rate of 1 l per 10 m^2^ of the coastal area next to the floating dam (a strip 25 m wide and about 40 m long). These biopreparations contain about 80 species of bacteria - actinomycetes, yeasts, and fungi (Lactobacillus, Bifidobacterium, Pediococcus, Lactococcus, Streptococcus, Rhodopseudomonas, Aspergillus, Mucor, and Streptomyces), with 1 g of the preparation containing 10^8^ to 10^10^ colony forming units (CFU)^25^. In parallel, EM applications were carried out in the water in the form of Bokashi balls and a liquid solution on and below the water surface in a separate zone of the Turawa reservoir. In total, 350 l of microbiological preparation was used for all the mentioned applications. Details of the taxonomic composition and the individual steps of application in water have been described in previous publications^16,25^. The assumption of the method is to synergistically act on the soil and restore the appropriate ecological balance of the ecosystem through the dominance of effective species in the soil.

### Laboratory tests

The substrate samples collected in the field were cooled in sterile containers and transported protected from the sun. They were then characterized in a fresh state (without drying) in the Environmental Chemistry Research Laboratory of the Institute of Technology and Life Sciences in Falenty to the following extent, including microbiological analyses analogous to those for soils:

- Heterotrophic bacterial count (HBN) by deep plating on Bunt and Rovira agar medium with the addition of soil extract and cycloheximide (50 µg per cm^3^ after 2 days of incubation at 28 °C^49^;
- The number of microscopic fungi (MF) on Martin agar with streptomycin (50 µg per cm^3^, counted after 5 days of incubation at 28 °C^50^;
- Coliform Index (CI, the smallest volume of soil in which coliform bacteria occur) using the multi-tube fermentation method on Eijkman’s liquid medium^51^;
- Dehydrogenase activity (DHA, an indicator of microbiological activity of soils) using 2,3,5-triphenyl tetrazolium chloride (TTC), according to the Polish standard PN-ISO 23753-1^53,54^.

The average number of HBN and MF colonies was converted to the dry weight of the medium based on the dilution of the medium solution used and the moisture content of the medium sample.

### Data analysis

In the data analysis, the temporal and spatial variability of the results was checked, both in general (at the study points and within the measurement campaigns) and in detail (within the EM application in the substrate - comparison of the group of control points, i.e., 1 and 2, and the study points affected by the EM application, i.e., 3–8, as well as taking into account the application data and the changes in the values of the analyzed parameters). Statistical analyses were performed using the following software: IBM SPSS Statistics 26, Statistica 13, Origin 2024b, and Excel 2021. Maps were created in QGIS Desktop 3.34.9. The scope of the analysis was as follows (from general to specific):

1. Overall variability of results for parameters at research points

- Descriptive statistics for microbiological parameters of the substrate at the study sites, divided into the control group and the group with EM application - minimum, mean, median, maximum, standard deviation, correlation coefficient;

2) Checking the similarity and correlation of results^54–57^:

- Hierarchical cluster analysis (HCA) - for points and parameters as a whole as well as for HBN, MF, CI, and DHA; analysis to check grouping according to predefined criteria; Ward method and correlations (classification into clusters);
- Principal Component Analysis (PCA) - for scores and parameters to examine the variability of results in space, with each component representing different factors influencing it; PCA also included a scree plot, community analysis, and sampling adequacy (expressed by Bartlett’s test for sphericity and the Kaiser-Meyer-Olkin KMO index);
- Spearman correlation matrix for a non-linear distribution - the direction and strength of the correlation were analyzed for pairs of study points and parameters; the following scale for correlation strength was assumed: *R* > 0.7 - strong, *R* = 0.3–0.7 - moderate, *R* < 0.3 - weak;

3) Comparison of results at control points and with the EM application^58^:

- Mann-Whitney U-test - comparison of medians for two groups of data with a non-linear distribution; in this case, the control points (1 and 2) and the points covered by the EM application (3–8) were considered;
- Boxplots - show graphically the variability of the results for soil microbiological parameters in two groups of points (control and with EM application), divided into 2019 and 2020;

4) Analyses checking the effectiveness of EM applications along with comparison with the literature:

- Line diagrams for the period under investigation at all measuring points with marked EM application data - verification of the potential effects on the values of the microbiological parameters;
- Effect size after and before EM application in soil for the four tested microbiological parameters - comparison of the results of the next measurement campaigns before and after EM application (1–16/09/2019 and 02/07/2019; 30 days after EM application, 2–28/07/2020 and 20/11/2019; 30 days after EM application, 3–23/11/2020 and 28/07/2020; 30 and 90 days after EM application).

## Results

### Overall variability of results for parameters at research points

The mean variability in the study points for HBN shows that the values in points 7 and 8 were higher than in the other points - by up to 181% (the difference between points 8 and 4). If you arrange these points in descending order, the median is as follows: 8 > 7 > 5 > 6 > 1 > 2 > 3 > 4. The difference between the maximum and minimum value of HBN ranged from 31.96 million CFU/g (point 4) to 49.94 million CFU/g (point 3). It is worth noting that the coefficient of variance indicates a high variability of values for this parameter (CV > 100%, in this case, from 166.53% in point 6 to 204.57% in point 3). Table 1 contains descriptive statistics for all microbiological parameters analyzed.

Different results were obtained for MF - in the case of the median in item 3, it was significantly higher than in the other items, even 195% higher than in item 6. The median values from the highest to the lowest in the items are: 3 > 5 > 4 > 2 > 1 > 8 > 7 > 6. The amplitudes ranged from 1725 CFU/g (point 8) to 3800 CFU/g (point 7). The CV is lower than for HBN, but its values are still high, i.e., above 100% (from 109.56% in point 3 to 150.34% in point 4).

The CI results indicate a very low number of coliform bacteria in the samples tested - a maximum of 0.01 g substrate in all points except 6 and 0.001 g in point 6. The medians were 0.0001 g (points 2, 4–6) or 0.001 g (points 1 and 3). The difference between the minimum and maximum values was 0.009 g to 0.0099 g. The CV values ranged from 45.44% (points 1 and 3) to 161.26% (point 7) - i.e., they were medium (CV between 30 and 100%) or high.

The last parameter, i.e., DHA, showed the smallest differences between the items, as shown by the low CV values (from 12.13% in item 5 to 26.95% in item 8). The medians differed by a maximum of 36.11% (points 4 and 7, 49 and 36 mg TPF/kg·h, respectively). If you arrange the points in descending order, the following order results: 4 > 5, 2 > 6 > 1 > 8 > 7, 3. The DHA amplitude was the same, from 17 mg TPF/kg·h (points 1 and 5) to 36 mg TPF/kg·h (point 8).

### Checking the similarity and correlation of results

#### HCA

**Fig. 2:**
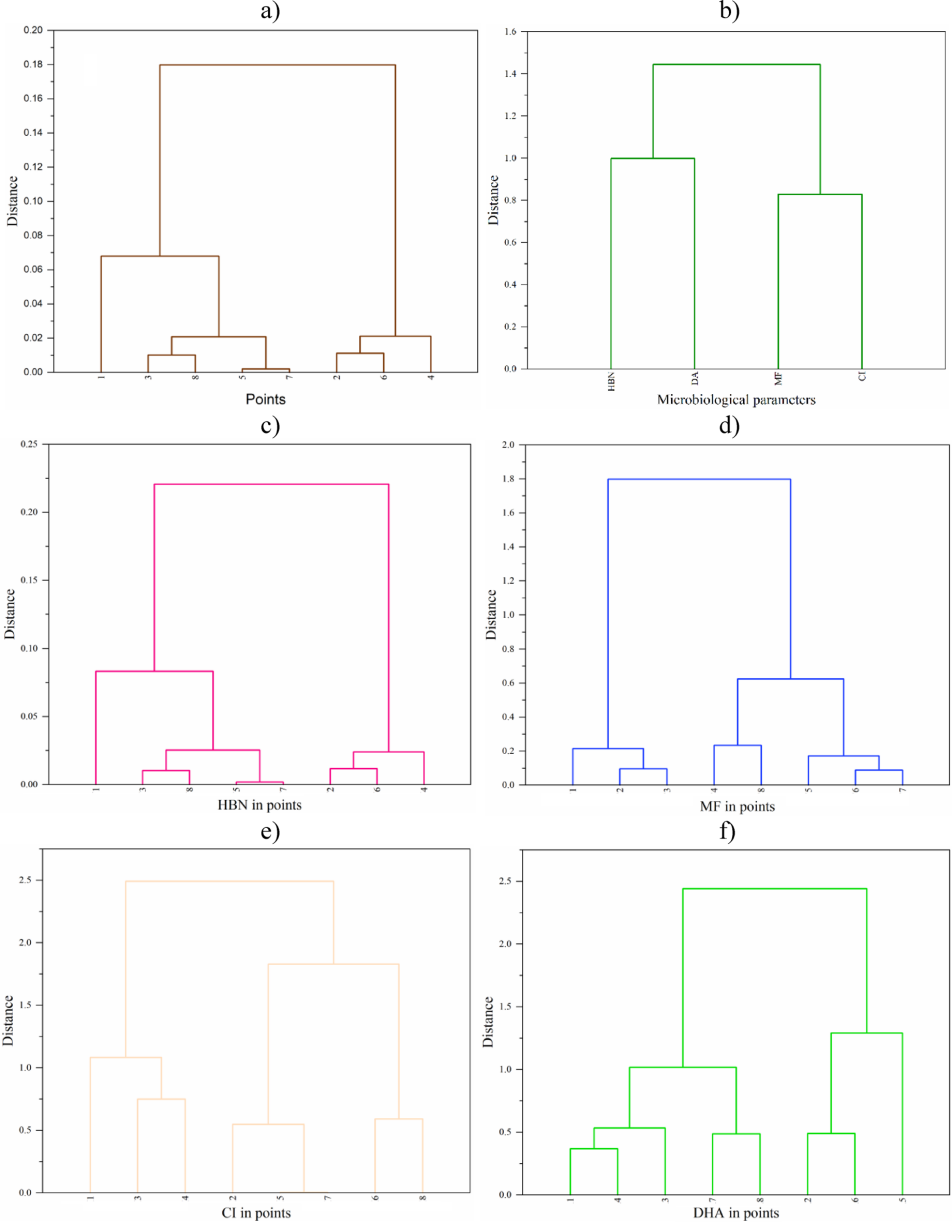
Hierarchical cluster analysis (HCA) for the results of substrates covered by EM within the Turawa reservoir in Poland: (a) research points, (b) microbiological parameters, (c) HBN, (d) MF, (e) CI, and (f) DHA.

The dendrograms resulting from the HCA are shown in Fig. 2A-F. As a result of splitting the whole dataset into points (Fig. 2A), it can be seen that cluster 1 contains five research points, namely 1, 3, 8, 5, and 7, while the second cluster contains three research points, namely 2, 6, and 4. Thus, they are different from the split into control points (1 and 2) and research points covered by the EM application (3–8). The parameters, on the other hand, are completely different if a distance of 0.8 or less is assumed as the location of the intersection. In the presented dendrogram (Fig. 2B), cluster 1 contains HBN and DHA, while cluster 2 contains MF and CI. To determine the grouping of points more precisely, analyses were also performed separately for each of the parameters (Fig. 2C-F). There is a large variability in the distinguished clusters with respect to the points analyzed, and their most similar feature is the independent occurrence of point 1 within the most detailed clusters (exception: DHA, where this point is in the same cluster as point 4). This confirms the different behavior of the microbiological parameters in the studied points in terms of their spatial and temporal variability, as well as the lack of a clear similarity for the control points and with the EM application, which also results from the general HCA for the whole data set in points.

**Table 1:** Descriptive statistics for Microbiological parameters in the substrate within the areas of application of effective microorganisms in the Turawa reservoir in Poland.

Group	Point	Min	Mean	Max	SD	Median	CV
Heterotrophic bacteria number (HBN, CFU/g)							
Control	1	2450	696,090	3,955,000	1331055.8	63,500	191.22%
	2	2450	738,530	3,935,000	1426779.4	56,500	193.19%
EM	3	1100	793,703	4,995,000	1623690.8	51,750	204.57%
	4	3900	530,800	3,200,000	1069859.5	50,100	201.56%
	5	24,400	760,177	4,100,000	1419823.8	79,000	186.78%
	6	38,100	734,177	3,580,000	1,222,611	70,500	166.53%
	7	41,100	880,153	4,370,000	1513709.5	129,500	171.98%
	8	12,900	749,400	3,897,000	1313023.7	141,000	175.21%
Microscopic fungi (MF, CFU/g)							
Control	1	42	977.13	3100	1143.2591	170	117.00%
	2	30	731.87	2700	906.40072	195	123.85%
EM	3	35	804.13	2540	880.96715	325	109.56%
	4	48	497.33	2700	747.69891	200	150.34%
	5	80	1050.13	3320	1200.4651	210	114.32%
	6	18	842.13	2320	1008.5111	110	119.76%
	7	40	991.13	3840	1251.7955	155	126.30%
	8	25	461.13	1750	535.74152	165	116.18%
Coliform index (CI, g)							
Control	1	0.001	0.0082	0.01	0.00373	0.01	45.44%
	2	0.0001	0.00442	0.01	0.00473	0.001	106.97%
EM	3	0.001	0.0082	0.01	0.00373	0.01	45.44%
	4	0.001	0.0028	0.01	0.00373	0.001	133.08%
	5	0.001	0.0028	0.01	0.00373	0.001	133.08%
	6	0.0001	0.00064	0.001	0.00046	0.001	71.31%
	7	0.0001	0.00244	0.01	0.00393	0.001	161.26%
	8	0.001	0.0028	0.01	0.00373	0.001	133.08%
Dehydrogenase activity (DHA, mg TPF/kg*h)							
Control	1	33	41.1333	50	5.22175	41	12.70%
	2	39	46.0667	59	6.48588	45	14.08%
EM	3	28	37.2667	50	6.29588	38	16.89%
	4	33	47.7333	61	9.30028	49	19.48%
	5	38	45.8	55	5.55749	45	12.13%
	6	31	41.7333	51	5.45719	42	13.08%
	7	28	36.8	48	5.58314	36	15.17%
	8	22	40.5333	58	10.92093	38	26.94%

#### PCA

**Fig. 3:**
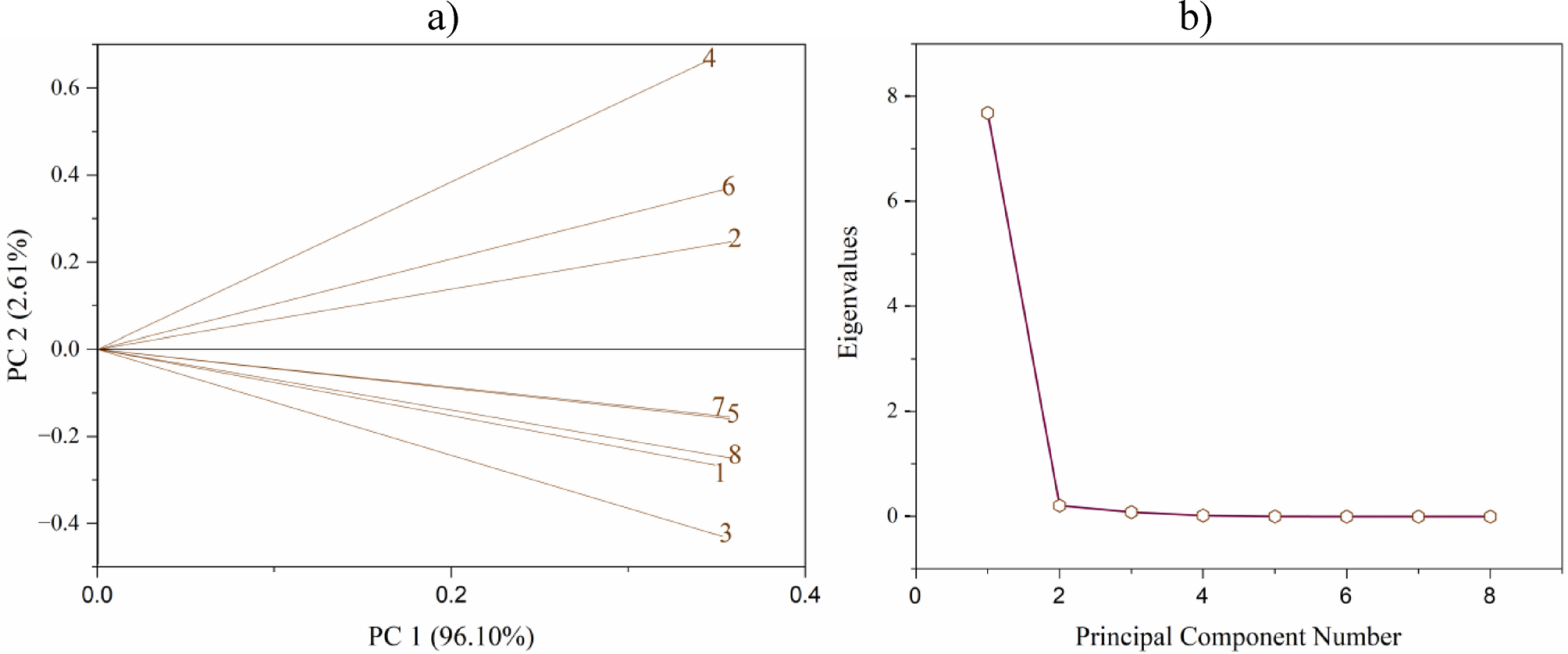
Principal component analysis (PCA) for the research points covered by the EM application in the substrate within the Turawa reservoir: (a) loading plot, (b) scree plot.

PCA was performed for each point and parameter. The KMO scores indicate sufficient sample adequacy for the analysis (or, according to Kaiser, substantial; a value equal to 0.807). In addition, Bartlett’s test for sphericity showed the statistical significance of the results (χ^2^ = 2268.912, d_f_ = 28, *p* < 0.0001). The analyzed points were very similar to each other, and all of them could be explained by one principal component (PC1, eigenvalue > 1), accounting for 96.1% of the explanations (Fig. 3). The largest difference between the values of the loading plots was observed between points 8 and 4, which was minimal, namely 0.01442 with respect to the x-axis (4.2%).

**Fig. 4:**
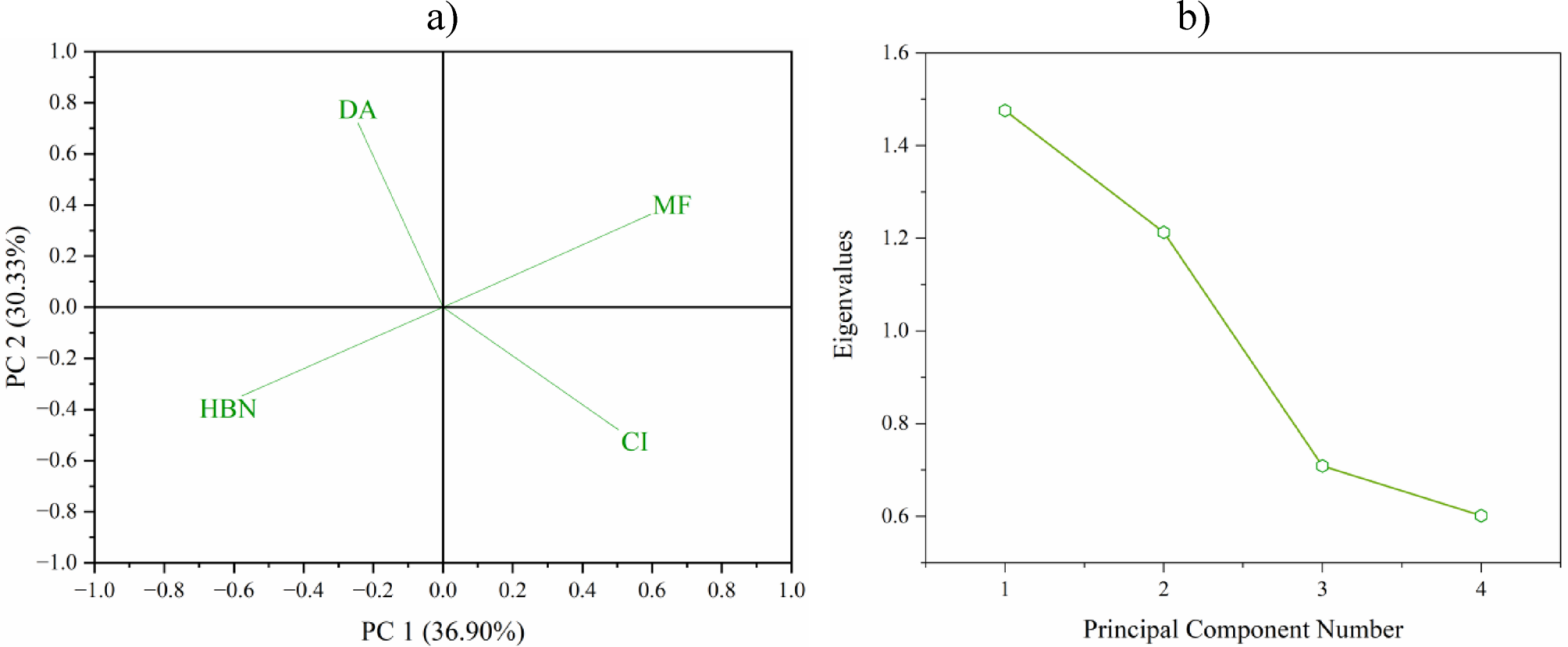
Principal component analysis (PCA) for microbiological parameters in the research points covered by the EM application in the substrate within the Turawa reservoir: (a) loading plot, (b) scree plot.

Broken down by parameter, the KMO value indicates lower sampling adequacy but is still sufficient to perform a PCA, i.e., higher than 0.5 (value 0.509). An additional analysis of the commonalities for the parameters showed that each of them can be considered in PCA after extraction (HBN = 0.637, MF = 0.684, CI = 0.652, DHA = 0.716). Bartlett’s test for sphericity was statistically significant (χ^2^ = 31.441, d_f_ = 6, *p* < 0.0001). In this case, the parameters were influenced by more factors. They differed from each other, as indicated both by their different location on the loading plot (each parameter in a different quarter of the graph) and by the two extracted principal components, which together explain 67.23% of the variability of the results (PC1 = 36.9%, PC2 = 30.33%). Figure 4 shows the dependencies described.

#### Spearman correlation matrix

**Fig. 5:**
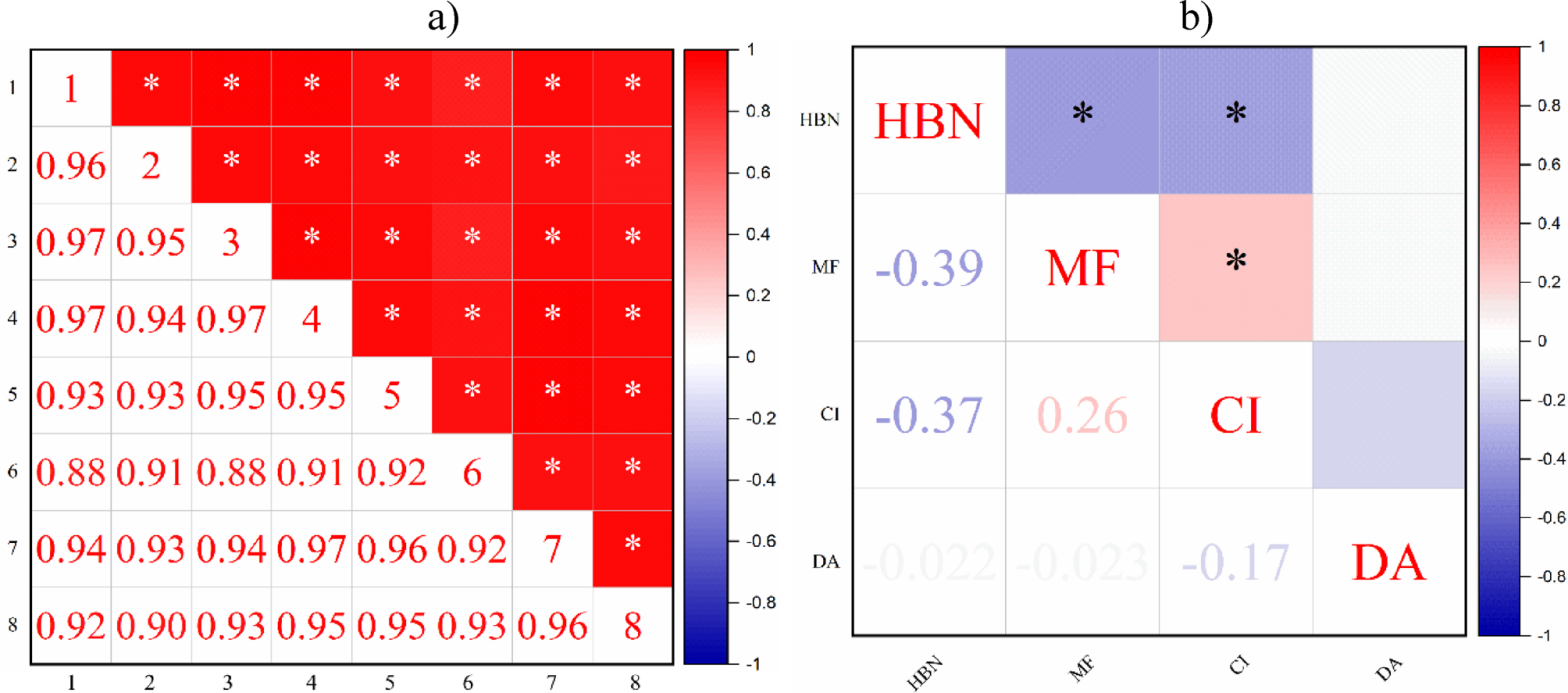
Spearman correlation matrix for substrate test results within the EM application for: (a) test points, (b) microbiological parameters.

The final analysis to determine the variability of the results was the Spearman correlation matrix for points and parameters. It confirms the results of the HCA and PCA, i.e. all points are strongly correlated (R > | 0.7 | and statistical significance for *p* < 0.05, values from 0.88 for points 1 and 6 and 3 and 6 to 0.97 for items 1 and 3, 1 and 4, 3 and 4, and 4 and 7), and the parameters are not as strongly correlated (statistical significance for MF and HBN = −0.39, HBN and CI = −0.37, and MF and CI = 0.26, i.e., medium and weak correlation strength - *R* = 0.3–0.7 and *R* < 0.3). Figure 5 shows the relationships in detail.

### Comparison of results at control points and with the EM application

#### Mann-Whitney U test

The non-parametric Mann-Whitney U-rank test was performed to verify whether the conclusions previously formulated on the basis of various statistical analyses regarding the absence of major differences between the control points (1–2) and the points covered by the EM application (3–8) were correct (Table 2). For the parameters analyzed, no statistically significant differences were found between the groups of points considered in 3 out of 4 cases. The exception is the CI, for which the z-measure was − 2.56061 and *p* = 0.01046. The mean and median values differ significantly in this case (mean − 0.00631 g for the control and 0.00328 g for the EM application, median − 0.01 g and 0.001 g), but these are microscopic amounts of the substrate in which coliform bacteria are present. Hence, these differences are not of great significance for the overall state of the substrate. Nevertheless, it should be emphasized that the coliform bacteria were generally less abundant at the sites covered by the EM application than at the control sites.

**Table 2:** Comparison of the results of Microbiological parameters of the substrate within the Turawa reservoir in control points (1–2) with points covered by the EM application (3–8) - Mann-Whitney U test.

Description	Microbiological parameters				
	Whole	HBN	MF	CI	DHA
U-value	21,102	1183.5	1331.5	927	1136.5
z-score	−0.37807	1.00606	−0.10909	−2.56061*	−1.29091
p	0.70394	0.3125	0.9124	0.01046	0.19706

Designations in the table: HBN - heterotrophic bacteria number, MF - microscopic fungi, CI - coliform index, DHA - dehydrogenase activity, * – statistically significant value for *p* ≤ 0.05.

#### Boxplots by point groups and years

**Fig. 6:**
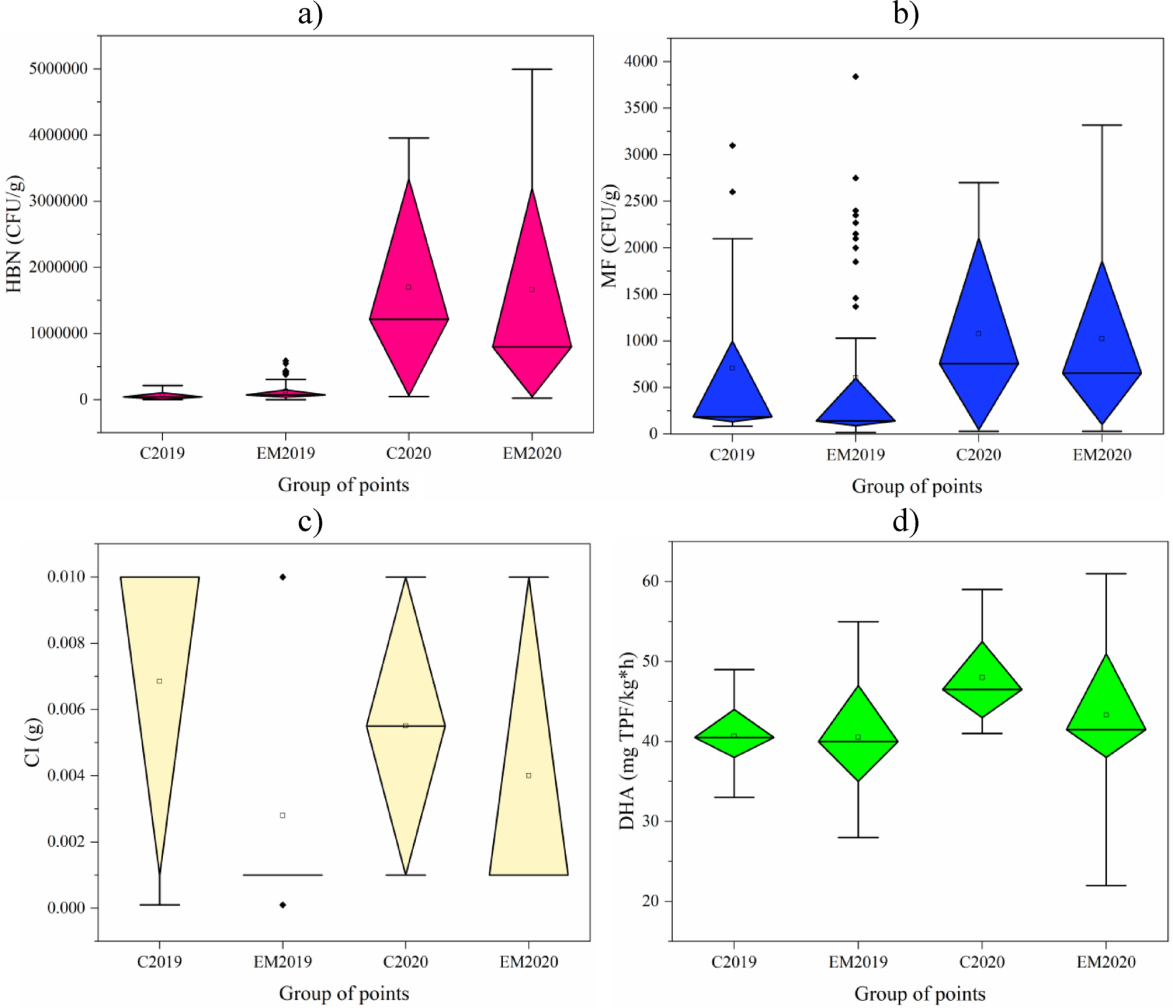
Results of substrate parameter tests near the Turawa reservoir for control points (C) and with the use of effective microorganisms (EM) in 2019 and 2020: (a) HBN, (b) MF, (c) CI, (d) DHA.

By analyzing in more detail the variability of the results obtained, i.e., by years and parameters in groups of points, it is also possible to verify their evolution in 2019 and 2020 (Fig. 6). In the case of the HBN, a large difference can be seen between the two years, which is greater than between the point groups themselves (Fig. 6A). For the median, this difference was 28-fold between the control points from year to year (1.174 M CFU/g) and 10-fold between the points with EM application (0.728 M CFU/g); for the same approach between EM application and control, it was 68.13% in 2019 (28,550 CFU/g) and − 34.36% in 2020 (−417,925 CFU/g), so the number of this group of microorganisms changed between the groups.

As for MF, their number was higher in 2020 than in 2019 in relation to the median value, as was the case for HBN, and this variability was greater year-on-year than when comparing groups of points with each other (309.46% for the control and 366.19% for EM). However, a convergence of results is observed when the group with EM application is compared with the control group (decrease in the median number of MF with EM application - by 24.05% in 2019 and 13.53% in 2020, i.e., −44.5 CFU/g, and − 102.5 CFU/g). From this, the preliminary conclusion can be drawn that the EM application causes a reduction in the number of MF in the substrate, which is verified in Sect. 3.4.

For CI, a visible relationship is a decrease in the value of this parameter in the group of points after EM application compared to the control - the median in 2019 was lower by 90.00% (−0.009 g) and in 2020 - by 81.82% (−0.001 g). Broken down by year within these groups of points, the median for the points captured by the EM application did not differ and was 45.00% lower for the control points in 2020 compared to 2019.

The last parameter, i.e., DHA, also shows a decrease in the value of this parameter in the EM group compared to the control group - by 1.23% in 2019 and by 10.75% in 2020 (respectively − 0.5 and − 5.0 mg TPF/kg·h). The year-to-year differences in medians were 14.85% for the control and 3.75% for EM (i.e., higher values in 2020 than in 2019). In this case, the variability of medians across years and within groups is comparable for this parameter.

### Analyses to check the effectiveness of effective microorganisms

**Fig. 7:**
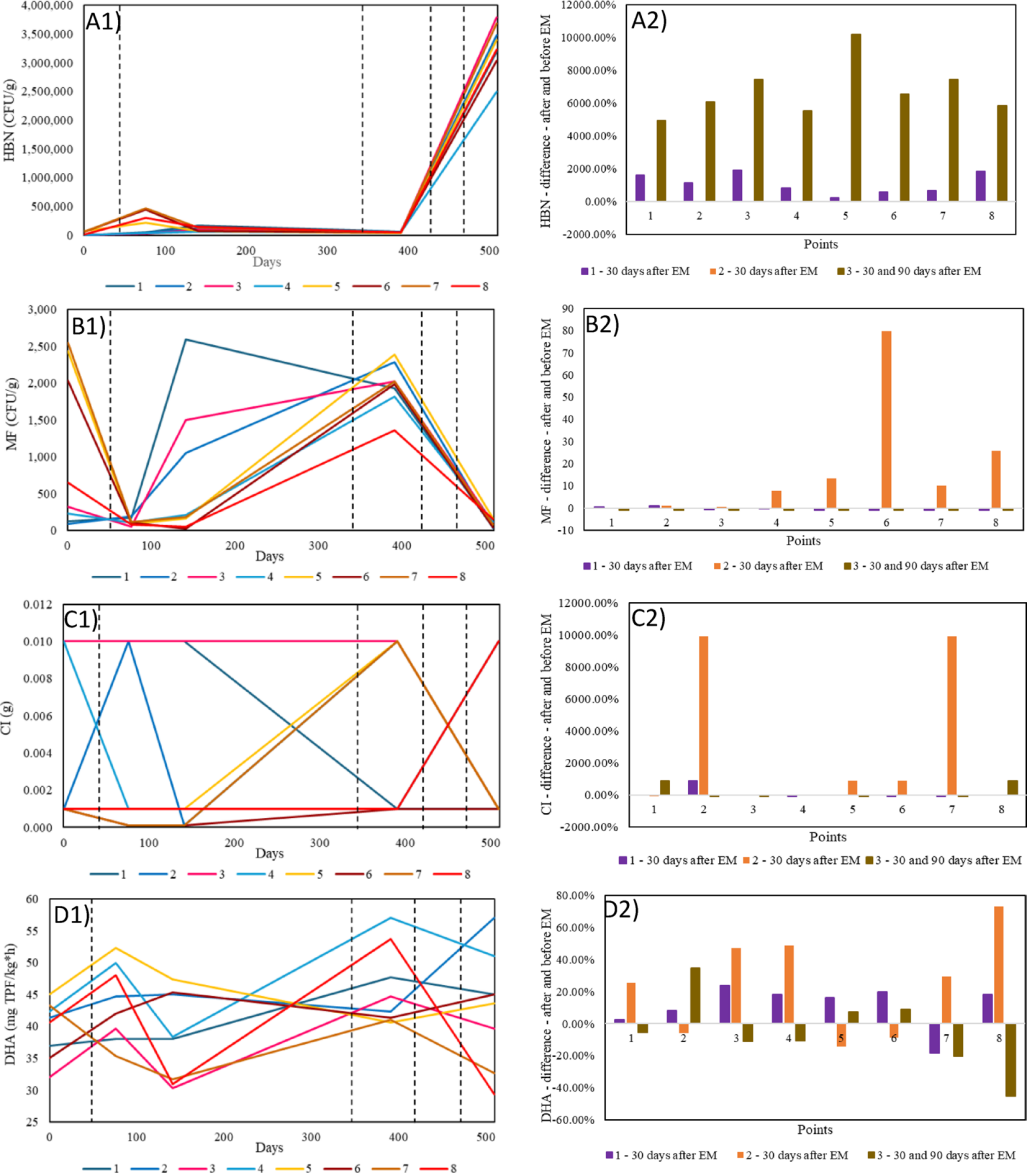
Variability of results with respect to the dates of EM application to the substrate (A1-D1) and the difference before and after EM application (A2-D2) for (a) HBN, (b) MF,.

The last element of the article is the verification of the effectiveness of EM in terms of the formation of microbiological parameters at all the points tested, taking into account the times of EM application in the substrate and the water of the Turawa reservoir (Fig. 7). The differences between the results for three EM applications were analyzed, namely: 30 days after EM application in August 2019 (1 - September 2019), 30 days after application in June 2020 (2 - July 2020), and 30 and 90 days after application in August and October 2020 (3 - November 2020). The closest previous measurement series was considered a zero date (1 - July 2019, 2 - November 2019, 3 - July 2020).

With regard to HBN (Fig. 7A), the results at all points of the individual measurement series show the same direction of change in the value of this parameter, but for rows 1 and 3 in a positive direction and row 2 - in a negative direction, which means that the number of soil bacteria has changed despite the application of EM. Looking at the averaged values of the effects in the individual groups, these changes are also ambiguous - for periods 2 and 3, the HBN was higher in the points with EM application than in the control points (2 - by 7.89%, 3 - by 1649.33%), while for period 1 - it was lower (by 361.66%). Thus, the behavior of the bacteria was convergent in all points, regardless of the application of EM. At the same time, the application of EM did not lead to a significant increase or decrease in points. It should be added that in the last series of measurements, i.e., after two EM applications, there was a significant increase in the average HBN, but this was visible in all points, including the control points, so it is not possible to draw a clear conclusion that EM application caused this.

In MF (Fig. 7b), in contrast to HBN, the trends of the changes in the points in the analyzed series are no longer uniform. In series 1, an increase in value was observed in points 1 and 2; in the others - a decrease; in series 2 - a decrease in value in point 1; and in the remaining points - an increase; in series 3 - a decrease in value in all points. In this case, the differences between the groups of control points and points with EM application were found entirely in the first term and the second term. It is worth mentioning that the averaged differences in series 2 were definitely the highest in point 6 (7965.56%) compared to the others (values from − 25.51% in point 1 to 2590.79% in point 8). Series 1 can be considered the most representative compared to the others, as no EM was applied on the first date in this interval (July 2019) and on the second − 30 days after EM application (September 2019), while in the other two series either the period was long and other factors could have been at work (November 2019 - July 2020, EM application in June 2020) or other EM applications have already been performed several times and it is difficult to determine the reference point for the substrate unchanged by EM (July 2020 - November 2020, EM application in June, August and October 2020). The averaged effects of MF for the control groups were: 1–74.72%, 2–45.97%, and 3 - −97.92%, while for the groups with EM application: 1 - −84.81%, 2–2235.04% and 3–4.10%. Considering the specificity of the studies conducted, it can be concluded that EM application may have had an impact on the reduction in the number of MF when considering the first group of studies.

The third parameter, i.e., CI (Fig. 7c), does not clearly show the influence of EM application on its formation - in series 1 and 3, there was a decrease in the value in this group of points, while in series 2 - an increase. Moreover, there was no convergence in the direction of changes between the groups of points, nor within the control point group itself (simultaneous decrease, increase, or no change in all series). Considering the first series as the most representative, the group with EM application showed a decrease in score by 90 (points 4, 6, and 7) or no change (points 3, 5, and 8), while the control group showed an increase or no change. The greatest difference was observed in the second series for points 2 and 7, with a 99-fold increase. For this parameter, it is also not possible to clearly determine the influence of the EM application on the formation of its value from a long-term perspective. If the first series is considered representative, EM causes a decrease in the CI value, which is also confirmed by the statistical significance in the Mann-Whitney U test.

The direction of the changes in the DHA values (Fig. 7D) differs both when comparing the series with each other and with the points themselves. In this case, there are also simultaneous decreases and increases within the compared study groups (in series 1, a negative value appears at point 7, while it is positive at the other points; in series 2, a decrease was observed at points 2, 5 and 6 and an increase in the value at the other points; in series 3, a negative value at points 1, 3, 4, 7 and 8 and at the other points - positive). The average values within the groups are as follows for the individual series in the control points and with the EM application, respectively: 1–5.38% and 12.99%, 2–9.76% and 29.27%, 14.53% and − 11.86%. When these values are compared with other parameters, they are significantly lower. DHA can be treated for this analysis as a parameter subject to changes that are difficult to predict and independent of EM application (even series 1, treated as representative, did not show specific relationships).

## Discussion

Table 3 shows a comparison of this study’s results with those of other studies in relation to the effectiveness of EM application for the microbiological parameters analyzed in the soil or substrate (except for CI, for which no publications on this medium were found). Most of them are consistent with the comparison of results for control points and those covered by EM application for HBN and MF - in both cases, the number of these groups of organisms generally increased after the application of the preparation (exception: HBN and MF - Frąszczak et al., 2012^63^, MF - Borowiak et al., 2021^65^). For DHA, the results are not clear, and after EM application, the values of this parameter decreased or increased, depending on the study.

**Table 3:** Comparison of mean results of Microbiological parameters (HBN, MF, DHA) in control soils/substrates and after EM application obtained by different researchers.

Parameter	Control	Appl. of EM	Reference
HBN (10^6^ CFU/g)	0.0614^a^	0.07045^a^	This study
	4.78	6.93	Higa and Wididana, 1991^60^
	5.477	13.962	Kaczmarek et al., 2008^61^
	30.93^b^	50.3^b^	Ghazal et al., 2011^62^
	10.32^b^	5.34^b^	Frąszczak et al., 2012^63^
	1.2^c^	6.033^c^	Fan et al., 2016^37^
	~ 2000	~ 5000	Du et al., 2020^64^
	~ 0.0000683	~ 0.000205	Borowiak et al., 2021^65^
	27.0	38.3	Bauza-Kaszewska et al., 2022^66^
	27.8	28.3	Breza-Boruta and Bauza-Kaszewska, 2023^67^
MF (10^3^ CFU/g)	0.185^a^	0.205^a^	This research
	9.42	39.1	Higa and Wididana, 1991^60^
	117.1	198.1	Kaczmarek et al., 2008^61^
	1.513	3.02	Ghazal et al., 2011^62^
	3530	1090	Frąszczak et al., 2012^63^
	~ 105	~ 175	Fan et al., 2016^37^
	~ 3.93	~ 3.85	Borowiak et al., 2021^65^
DHA (mg TPF/kg·h)	41.5^a^	40.5^a^	This research
	~ 73.0	~ 65.0	Park and Kremer, 2007^68^
	7.89	7.57^d^, 8.01^e^	Mayer et al., 2010^69^
	~ 9.0	~ 10.8	Tang et al., 2010^70^
	16.36	26.80	Ghazal et al., 2011^62^
	7.64^f^, 2.72^g^	9.92^f^, 2.86^g^	Sharma et al., 2017^71^
	~ 0.0175	~ 0.0142	Borowiak et al., 2021^65^
	~ 86.0	~ 113.0	Elbagory, 2023^72^
	~ 200.0	~ 314	Li et al., 2023^42^

a - median value, b - total bacterial counts (TBN), c - in 4 out of 5 series similar results in both groups, resulting from the last series, d - EM spraying, e - EM spraying + Bokashi balls, f - Calendula field, g - Marigold field, variability from 270 to 350 (*Saccharomyces cerevisiae*,* Rhodotorula benthica*,* Lactobacillus plantarum*,* Bacillus thuringiensis*), ~ - approximate values, read from the charts.

Regarding the magnitude of changes, they ranged for HBN from − 48.26% (Frąszczak et al., 2012)^63^ to 402.75% (Fan et al., 2016)^37^ (mean = 102.56%, median = 53.80%; 9 out of 10 studies demonstrated an increase in HBN after EM application). For MF, the range was from − 69.12% (Frąszczak et al., 2012)^54^ to 315.07% (Higa and Wididana, 1991)^59^ (mean = 70.02%; median = 66.67%; positive direction of change in 5 of 7 cited studies). The last parameter, DHA, ranged from − 18.86% (Borowiak et al., 2021)^65^ to 63.81% (Ghazal et al., 2011)^61^ (mean = 15.68%, median = 5.15%, increase in DHA after EM application in 8 of 11 sources, with two studies dividing the results into two different cases). The results presented in this paper for HBN and MF agree with most of the cited sources, while in the case of DHA - they differed, but the difference after EM application was only − 2.41%, which is below the most commonly accepted statistical error of 5%. Differences in the absolute values of the parameters may be due to the soil and climatic conditions or the way the study was conducted (e.g., the conditions of EM application, its dose, and form).

The studies cited in Table 3 generally focused on testing the suitability of EM as a fertilizer to improve crop production (wheat, corn, kiwi, *Salvia splendens*, Calendula, Marigold, *Salvia coccinea*), often in comparison with other soil fertility improvers (poultry manure, straw, agar, starch, compost, liquid fertilizer). The vast majority were conducted under field conditions. The second group concerned studies related to soil remediation - in this case, the removal of hydrocarbons and petroleum derivatives in industrial areas^42,69^, the desalination of agricultural soils^71^, and the improvement of the physicochemical and microbiological properties of soils of degraded mountain pastures^43^. In all studies, relevant analyses of soil properties, including HBN, MF, and/or DH, were also performed.

Higa carried out the first studies on EM. In the cited results from 1988^60^, the plate method was used to examine gray mountain soil with regard to the change in its properties and the yield of spinach and tomato with different variants of soil use (control, fertilization, and EM applications in various forms, including the well-known liquid form with a mixture of 80 species of coexisting microorganisms). The results showed that the mixture used caused an increase in HBN and MF compared to the control sample, but also in relation to the sample with fertilization. However, the HBN for the described solution was lower than for the two other types of EM tested (with photosynthetic bacteria in a liquid medium and with the genus Lactobacillus and lactic acid-producing microorganisms). In the case of MF, they were the most numerous among the proposed variants in the preparation. However, it is important to note that the tomato yield after the application of EM was lower than in the test with fertilizers (but higher than in the control).

In comparison, the spinach yield was higher (also than in the control). As emphasized, the effect of EM promotes more effective root penetration into the soil and an improvement of its utilization properties - bacteria help in the aggregation and synthesis of soil binders and fungi - in the binding of soil particles into more stable aggregates. Ultimately, EM was introduced to the market as a mixture of microorganisms belonging to bacteria, fungi, and actinomycetes, and it was in this form that it was mainly tested in the following years.

As already mentioned, most of the studies cited indicate an increase in HBN and MF after the addition of EM. However, Frąszczak et al. (2012)^62^ came to different results for both parameters than in most studies. The authors point out that the decrease in HBN after EM application was influenced by the utilization of nutrients in the soil by microorganisms from EM as well as by food competition from other microorganisms in the soil (in the seed samples also studied by the influence of auxins and gibberellins secreted by them, which can inhibit the development of some microorganisms). It is also worth mentioning that the studies were carried out under controlled conditions, i.e., in plant chambers with a peat substrate, which could have influenced the results obtained. Another factor could be the material used, as stated by Priyadi et al. (2005)^72^. They explained that the reason for the small difference in the results after using EM in combination with HBN and MF was that the original soils from wetlands (Inceptisols and Histosols) had a higher content of bacteria and fungi than EM.

According to the results of the studies, DHA generally increased after the addition of EM, i.e., it stimulated the general microbiological activity of the soil. An interesting case is a study by Mayer et al. (2010)^68^ on the electric medium Regosol (sandy loam) in Switzerland, where this parameter decreased or increased depending on the form of EM application. In the first case, a liquid application (spraying of EM) was used, while in the second case, this method was combined with the application of Bokashi balls. A general conclusion was drawn about the low effectiveness of EM on soil quality, while the slightly higher effect of Bokashi balls was due to the nutrient input they provided. Park and Kremer (2007)^67^ pointed to the selective inhibition or stimulation of certain components of the microbiological community as the cause of the variability in DHA results after EM application. The effect of EM also depended on the organic material with which it was applied, i.e., the interaction with it (on Mexico silt loam in the United States, EM was tested with compost, poultry litter, and straw).

In summary, EM has the potential to improve the microbiological activity of the soil/substrate and increase plant yields. In relation to soil, reports also describe them as limiting decay processes, accelerating metabolism, increasing photosynthesis, increasing humus content, detoxifying pesticide-contaminated soils, and inhibiting the development of plant pathogens68. Therefore, there is a potential for bioremediation of the tested substrate in the eutrophic Turawa reservoir. However, the effectiveness of EM in liquid form and with Bokashi balls was found to be significantly higher in improving water quality in terms of microbiology and trophic levels14. As other studies have shown, the effects of EM include immobilization of nitrogen, easy mineralization of the pool of organic matter^73^, increased concentration of organic carbon, total nitrogen, and available phosphorus^43^, increased biodiversity and decomposition of organic matter, which can promote crop growth, among other benefits^10^. Also of importance in the context of eutrophic systems are questions about the relationship between nutrients and chlorophyll a and other factors^74^, including modeling changes in chlorophyll a in this type of ecosystem^75^.

The literature analysis has shown that the application of EM to HBN, MF, and DHA generally leads to an increase in the values of these parameters. The results obtained in terms of medians for the control point groups and with the application of EM were consistent with respect to HBN and MF. For DHA, however, they differed from most studies (although the difference was only marginal at 2.41%). Any decreases after the application of EM are due to a higher number of microorganisms in the control soils than in the preparation, to the specific action of other soil organisms, or to the secretion of toxins by the seeds that inhibit the development of these groups of organisms. The studies focused mainly on the use of EM as a means to stimulate the development and growth of plants and to remediate contaminated soils or soils with unfavorable use characteristics.

The decrease in the CI value after the application of EM means that the number of these bacteria decreased after the application of EM. Thus, it can be assumed that the oxygen conditions in the tested medium could have improved^76^. The described bacteria are a problem, especially for sanitary reasons (fecal contamination) and negative health implications (enteropathogens). In addition, increased concentrations of *Escherichia coli* can inhibit the removal of nutrients (especially total nitrogen and ammonium), as well as inhibit bacterial genetic metabolic pathways^77^. Their sources are mainly related to anthropogenic activities, i.e., industrial sewage discharges, runoff from agricultural fields, non-point anthropogenic sources, and animal waste^78^. CI is, therefore, one of the indicators of the effectiveness of soil remediation in the coastal zone of a eutrophic reservoir.

The presented studies on EM in the Turawa reservoir substrates applied as a spray on the substrate surface showed a lower efficacy than EM in water applied in liquid form and as Bokashi balls. They can be treated as an auxiliary method for remediation. To clearly determine the effectiveness of these measures, it would be necessary to use different forms and dosages of EM in the substrate and to adjust the sampling data to the substrate application data. It would also be advisable to expand the scope of analyses in the future to include actinomycetes, which are an important component of EM preparation, and to perform microbiological analyses of taxonomic composition in samples of control substrates and those to which EM has been applied. To place the potential effects of EM in a broader context, other physicochemical analyses related to soil water capacity and granulometric composition should also be considered. It would also be useful to take soil samples in the entire profile and not only in the top layer, i.e., the substrate, as in the study described. In addition, research should also evaluate the influence of other environmental factors (e.g., temperature fluctuations, water flow, presence of other chemicals) on the results presented. To this end, one can try to calculate the fertility and toxicity indices in the substrate before and after the application of EM but also link them to the water quality results from other water bodies collected in databases, e.g., the one published in 2024, which includes almost 14,000 lakes in 77 different countries^79^.

## Conclusions

The statistical analyses performed (HCA, PCA, Spearman correlation matrix) showed that (1) there was no significant variability in the results between the study points analyzed (exception: HCA for 3 out of 4 parameters, where control point 1 was placed in a separate cluster); (2) each of the considered microbiological parameters in the medium (heterotrophic bacterial count - HBN, microscopic fungi - MF, coliform index - CI, dehydrogenase activity - DHA) were significantly different from each other; (3) the study points were statistically significant (*p* < 0.05) and strongly correlated (*R* > 0.7) - correlation strength from 0.88 to 0.97, while the parameters are not so strongly correlated (statistical significance for MF and HBN = −0.39, HBN and CI = −0.37 and MF and CI = 0.26, i.e. medium and weak correlation strength - *R* = 0.3–0.7 and *R* < 0.3).

When comparing the group of control points (1, 2) and the points covered by the application of effective EM microorganisms (3–8) using the Mann-Whitney U-test, statistically significantly different results were found only for CI - in this case, the mean and median values differed significantly (respectively: Mean − 0.00631 g for the control and 0.00328 g for the EM application, median − 0.01 g and 0.001 g). However, these are microscopic amounts of substrate in which coliform bacteria are present, so these differences will not have a major impact on the overall condition of the substrate. It should also be emphasized that coliform bacteria were generally less abundant at the sites covered with the EM application than at the control sites.

Considering the timing of the EM application, the results are ambiguous for the following parameters:

- For HBN, the results show the same direction of change in the value of this parameter in the individual series of measurements despite the application of EM (for series 1 and 3, it is a positive direction, and for series 2 - it is negative). Looking at the averaged values of the effects in the individual groups, these changes are also ambiguous - for periods 2 and 3, the HBN was higher in the points with EM application than in the control points (2 - by 7.89%, 3 - by 1649.33%), while for period 1 - it was lower (by 361.66%). Thus, the behavior of the bacteria was similar in all points, regardless of the application of EM, and at the same time, the application of EM did not lead to a clear increase or decrease in the points.
- In the case of MF, in contrast to HBN, the trends of changes in points in the analyzed series are no longer uniform (in series 1, an increase in the value for points 1 and 2 was observed; in the others - a decrease, in series 2 - a decrease in the value in point 1 and the remaining points - an increase, in series 3 - a decrease in the value in all points). The averaged MF effects for the control groups were: 1–74.72%, 2–45.97%, and 3 - −97.92%, while for the groups with EM application: 1 - −84.81%, 2–2235.04% and 3–4.10%. Considering the specificity of the studies conducted, it can be concluded that EM application may have had an impact on the reduction in the number of MF when considering the first group of studies.
- In the case of CI, it is not possible to clearly determine the effect of EM application on the formation of this parameter - in series 1 and 3, there was a decrease in the value in this group of points, while in seriess 2 - an increase. Moreover, there was no convergence in the direction of changes between the groups of points or within the group of control points itself. In series 1, there was a 90% decrease in value (points 4, 6, and 7) or no change (points 3, 5, and 8) after EM application, while in the control group there was an increase or no change. The highest difference observed was 99-fold in series 2, in points 2 and 7. Even for this parameter, it is not possible to clearly determine the effect of EM application on the long-term formation of the value. Suppose the first series is considered representative (based on the comparison of the substrate without EM application and one month after the first EM application). In that case, EM causes a decrease in the CI value, which is also confirmed by the statistical significance of the Mann-Whitney U-test. A decrease in CI means that the oxygen conditions in the substrate have probably improved, which is a positive effect for its remediation due to the proven negative effects of E. coli on human health, bacterial balance, and the possibility of nutrient enrichment in the substrate.
- The direction of the changes in the DHA values differed both when comparing the series with each other and with the points themselves. In this case, there are also simultaneous decreases and increases within the compared study groups. The average values within the groups are as follows for individual series in the control points and with the EM application, respectively: 1–5.38% and 12.99%, 2–9.76% and 29.27%, 14.53% and − 11.86%. If you compare these values with other parameters, they are significantly lower. DHA can be treated as a parameter that undergoes changes for this analysis that are difficult to predict independently of the EM application.

The results described represent one of the case studies on the bioremediation of substrates and the improvement of their functional properties, which are important both for food production and for ensuring adequate quality of soils threatened by a range of anthropogenic pressures. Soils and substrates themselves are also an important element in the provision of ecosystem services. Research also fits into strategies for rational soil management, which is governed by numerous national and international legal acts, strategies, and policies (e.g., in the context of the Sustainable Development Goals). As a future research direction, it would be worthwhile to analyze the influence of effective microorganisms on soil properties (e.g., physico-chemical, microbiological) in comparison to other remediation methods (e.g., biological, physical, chemical) on a broader time-space scale.

## Data Availability

The datasets used and/or analyzed during the current study are available from the corresponding author on reasonable request.
