# Author Correction: Application of effective microorganisms for Littoral zone restoration in eutrophic reservoirs

**DOI:** 10.1038/s41598-025-09187-5

**Published:** 2025-07-10

**Authors:** Paweł Tomczyk, Barbara Wróbel, Czesława Rosik-Dulewska, Alban Kuriqi, Mirosław Wiatkowski, Witold Skorulski, Tomasz Kabat, Mirosław Prycik, Jarosław Drobnik, Łukasz Gruss, Andrzej Kłos

**Affiliations:** 1 Institute of Environmental Engineering, Wrocław University of Environmental and Life Sciences, 50-363 Wrocław, Poland; 2 Institute of Technology and Life Sciences, National Research Institute, 05-090 Falenty, Poland; 3 Institute of Environmental Engineering of the Polish Academy of Sciences, Zabrze, 41-819 Zabrze, Poland; 4 Civil Engineering Department, University for Business and Technology (UBT), 10000 Pristina, Kosovo; 5 Art Strefa Witold Skorulski, 51-604 Wrocław, Poland; 6 DATII (Dolnośląski Akcelerator Technologii i Innowacji), Długołęka, Poland; 7 Department of Family Medicine, Wroclaw Medical University, Wrocław, Poland; 8 Institute of Environmental Engineering and Biotechnology, University of Opole, 45-032 Opole, Poland

Correction to: *Scientific Reports* 10.1038/s41598-025-01795-5, published online 14 May 2025

In the original version of this Article, Alban Kuriqi was incorrectly affiliated with ‘Faculty of Environmental Engineering, Wroclaw University of Science and Technology, 50-370 Wroclaw, Poland.’

The correct Affiliation is listed below:

Alban Kuriqi

- Civil Engineering Department, University for Business and Technology (UBT), 10000 Pristina, Kosovo.

The original Article has been corrected.

